# Targeting murine alveolar macrophages by the intratracheal administration of locked nucleic acid containing antisense oligonucleotides

**DOI:** 10.1080/10717544.2019.1648589

**Published:** 2019-08-06

**Authors:** Yasunori Uemura, Katsuya Kobayashi

**Affiliations:** Immunology & Allergy Research Laboratories, Immunology & Allergy R&D Unit, R&D Division, Kyowa Kirin Co., Ltd, Nagaizumi-cho, Japan

**Keywords:** Pulmonary delivery, antisense oligonucleotides, locked nucleic acid, alveolar macrophages

## Abstract

The pulmonary delivery of locked nucleic acid containing antisense oligonucleotides (LNA-ASOs) is expected to be a novel therapeutic approach for lung diseases. However, there are two concerns to be considered: immune responses, as the lung has a distinct immune mechanism to protect it from inhaled pathogens; and leakage into blood, since the lung is permeable to small molecules. As phagocytic alveolar macrophages reside in the alveolar space, it is hypothesized that inhaled LNA-ASOs effectively accumulate and exert a knockdown (KD) effect on these cells at low doses. Thus, targeting alveolar macrophages by inhaled LNA-ASOs may reduce these risks. To test this hypothesis, LNA-ASOs targeting Scarb1 or Hprt1 were intratracheally administered to mice. We confirmed the remarkable accumulation of intratracheally administered LNA-ASOs in murine alveolar macrophages and found that they exerted a significant and sequence-dependent KD effect. The dose required for KD in alveolar macrophages was lower than that required to induce KD in the whole lung. Furthermore, when a KD effect was observed in alveolar macrophages, no KD effect was observed in the liver or kidney; however, several inflammatory cytokines were increased in the lung. These results suggest the potential application of LNA-ASOs as an inhaled drug specific to alveolar macrophages. However, further studies on the immuno-stimulatory effects of LNA-ASOs will be necessary for the development of novel inhaled therapeutic agents.

## 1. Introduction

Antisense oligonucleotides (ASOs) are synthetic single-stranded strings of nucleic acids that bind to RNA (Bennett, 2019). ASOs bind complementary RNA by Watson–Crick base pairing and suppress gene expression (knockdown, KD). They have the potential to be a next-generation therapy because they can modulate molecules that cannot generally be targeted using small molecules or antibodies. In particular, locked nucleic acid containing ASOs (LNA-ASOs) are not only stable *in vivo* but also show a high binding affinity and specificity to the target RNA compared to conventional ASOs without any chemical modifications of their gap positions (Kauppinen et al., 2005; Veedu & Wengel, 2010; Hagedorn et al., 2018). Several reports have indicated that LNA-ASOs exerted a KD effect *in vivo* without any drug delivery system (Gupta et al., 2010; Straarup et al., 2010; Delgado et al., 2015; Morihara et al., 2017). However, after systemic administration, LNA-ASOs broadly accumulate in various tissues, which can increase the risk of side effects in peripheral tissues (Frazier, 2015). In this regard, pulmonary drug delivery has the potential to minimize systemic exposure and reduce the risk of side effects of LNA-ASOs.

However, two concerns have been raised regarding inhaled LNA-ASOs therapy focusing on the lung. The first concern involves the immune response in the lung. As the lung has distinct immune mechanisms to protect it against foreign substances (Lafferty et al., 2010; Opitz et al., 2010), inhaled LNA-ASOs have the potential to induce inflammation. In fact, our previous study confirmed that the intratracheal administration of LNA-ASOs exerted a KD effect in the lung but induced an inflammatory response (Uemura et al., 2017). The second concern involves leakage from the lung. Since the lung is permeable to small molecules (Patton et al., 2004; Smola et al., 2008), inhaled LNA-ASOs may leak into the blood and accumulate in other organs. Therefore, with the lung-specific treatment, the leakage of the LNA-ASOs from the lung is an issue. Since the systemic administration of ASOs is well known to exert a KD effect on the liver and kidney (Swayze et al., 2007; Donner et al., 2015; Shen & Corey, 2018), inhaled LNA-ASOs may also exert a KD effect on these organs. These concerns should be considered while developing novel inhaled LNA-ASOs for therapeutic applications.

In our previous report, we investigated a KD effect of intratracheally administered LNA-ASOs in the whole lung (Uemura et al., 2017). The dose required for KD was the same as that inducing a neutrophilic immune response. As the lung is composed of various types of cells, the dose required for KD likely differs among cells. If we could target one fraction of lung cells with a lower dose than would be required to achieve KD in the whole lung, both the immuno-stimulatory effect and the leakage of inhaled LNA-ASOs might be avoided. Since phagocytic alveolar macrophages are present in the alveolar space (Hussell & Bell, 2014; Kopf et al., 2015; Morales-Nebreda et al., 2015), inhaled LNA-ASOs may be expected to accumulate effectively in alveolar macrophages and exert a KD effect at low doses. In addition, these cells are involved in several pulmonary diseases. We therefore hypothesized that targeting alveolar macrophages with inhaled LNA-ASOs might be useful as a novel therapy for pulmonary diseases. To test this hypothesis, two types of LNA-ASOs targeting scavenger receptor BI (Scarb1) and hypoxanthine phosphoribosyltransferase 1 (Hprt1) were prepared (Scarb1-ASOs and Hprt1-ASOs). These molecules have been used as targets to investigate the *in vivo* KD effect of nucleic acids (Murray et al., 2012; Sugo et al., 2016).

In the present study, Scarb1-ASOs and Hprt1-ASOs were cultured with thioglycollate-elicited murine peritoneal macrophages to investigate a KD effect *in vitro*. Subsequently, fluorescence-labeled Scarb1-ASOs were intratracheally administered to mice and the distribution in the lung was examined. Then, after the intratracheal administration of LNA-ASOs to mice, a KD effect in alveolar macrophages and whole lung was analyzed. In addition, the immuno-stimulatory effect and leakage of the intratracheally administered LNA-ASOs were also investigated.

## 2. Materials and methods

### 2.1. Mice

Seven-week-old female C57BL/6 mice were purchased from Charles River Laboratories Japan, Inc. (Ibaraki, Japan), and experiments were performed when the mice were 8 weeks of age. All animal studies were performed in accordance with the Standards for Proper Conduct of Animal Experiments at Kyowa Kirin Co., Ltd. (Nagaizumi-cho, Japan) under the approval of the company’s Institutional Animal Care and Use Committee. Kyowa Kirin Co., Ltd. is fully accredited by the Association for the Assessment and Accreditation of Laboratory Animal Care, International.

### 2.2. Oligonucleotides

LNA-ASOs complementary to mouse Scarb1 or Hprt1 mRNA were chemically synthesized by GeneDesign, Inc. (Osaka, Japan).

The sequences of the LNA-ASOs were as follows.

AF647-conjugated Scarb1:

LT^∧^LC^∧^A^∧^G^∧^T^∧^C^∧^A^∧^T^∧^G^∧^A^∧^C^∧^T^∧^LT^∧^LC-Am-AF647
Scarb1: LT^∧^LC^∧^A^∧^G^∧^T^∧^C^∧^A^∧^T^∧^G^∧^A^∧^C^∧^T^∧^LT^∧^LC
Hprt-1: LA^∧^LT^∧^LA^∧^G^∧^G^∧^C^∧^T^∧^C^∧^A^∧^T^∧^A^∧^G^∧^T^∧^LG^∧^LC^∧^LA
A, T, G, C: DNA; ^∧^: phosphorothioate bond; LN: LNA; LC: LNA 5-Methyl cytosine; Am: ssH amino linker.

### 2.3. Thioglycollate-elicited murine peritoneal macrophages and culture

Thioglycollate-elicited murine peritoneal macrophages were harvested as described below (Uemura et al., 2019). In brief, 1 mL of 3% thioglycollate (Wako Junyaku, Osaka, Japan) was intraperitoneally administered. Three days after the administration, mice were euthanized, and the peritoneal cells were isolated by washing the peritoneal cavity with PBS (Thermo Fisher Scientific, Waltham, MA). To assess the uptake of LNA-ASOs by peritoneal macrophages *in vitro*, harvested peritoneal cells were seeded at 5 × 10^4^ cells/well in a 96-well flat-bottom plate, and non-adherent cells were removed after 2-h culture. Subsequently, adherent macrophages were cultured with 8, 40, or 200 pg/mL of AF647-conjugated Scarb1-ASOs for 2 h. To assess the *in vitro* KD effect, adherent peritoneal macrophages were cultured with 0.4, 2, 10, or 50 µg/mL of Scarb1-ASOs or HPRT1-ASOs for one day.

### 2.4. The intratracheal administration of LNA-ASOs

AF647-conjugated Scarb1-ASOs, Scarb1-ASOs, or Hprt1-ASOs were dissolved in PBS and applied by a MicroSprayer MS-IA-1C (Penn-Century, Wyndmoor, PA). AF647-conjugated Scarb1-ASOs were administered as a single dose of 0.5 µg in 50 μL of PBS. Scarb1-ASOs or Hprt1-ASOs were administered at doses of 5 μg or 20 μg per mouse in 50 μL of PBS. The control mice received 50 μL of PBS. LNA-ASOs or PBS were administered under light anesthesia with isoflurane. Two hours after the administration of AF647-conjugated Scarb1-ASOs, the lung was harvested for flow cytometry. One day after the administration of Scarb1-ASOs or Hprt1-ASOs, the bronchoalveolar cells, right lung, liver, or kidney was harvested for the mRNA expression analyses.

### 2.5. The preparation of primary mouse lung cells

Primary mouse lung cells were prepared using a gentleMACS Dissociator (Miltenyi Biotec, Bergisch Gladbach, Germany) with reference to the modified protocol of the Lung Dissociation Kit (Miltenyi Biotec, Bergisch Gladbach, Germany). In brief, mice were euthanized with CO_2_ in an appropriate chamber and the lungs were harvested. The lungs were digested by DMEM supplemented with penicillin–streptomycin (Thermo Fisher Scientific, Waltham, MA) and 1.5 mg/mL of collagenase A (Roche Diagnostics, Basel, Switzerland), and then the 37C_m_LDK_1 and m_lung_02 programs of the gentleMACS Dissociator were run. After digestion, the erythrocytes in the lung cell suspension were lysed with BD Pharm Lyse Lysing Solution (BD Biosciences, San Jose, CA).

### 2.6. Flow cytometry

The uptake of AF647-conjugated Scarb1-ASOs or the cell surface phenotypes of murine macrophages were analyzed by flow cytometry. Adhered thioglycollate-elicited murine peritoneal cells were suspended in FACS buffer of PBS with 2 mmol/L EDTA (Thermo Fisher Scientific, Waltham, MA) and 2% (v/v) FBS (Thermo Fisher Scientific, Waltham, MA). To investigate the surface marker, after 5-min incubation with Purified Rat Anti-Mouse CD16/CD32 (BD Biosciences, San Jose, CA), cells were incubated with a cocktail of monoclonal antibodies, which included anti-mouse CD11b FITC (BD Biosciences, San Jose, CA) and APC anti-mouse F4/80 (Thermo Fisher Scientific, Waltham, MA). As isotype controls of those antibodies, rat IgG2b κ isotype control FITC (BD Biosciences, San Jose, CA) and APC rat IgG2a κ isotype control (Thermo Fisher Scientific, Waltham, MA) were used. Thioglycollate-elicited murine peritoneal macrophages cultured with AF645-conjugated Scarb1-ASOs were suspended in FACS buffer.

Prepared primary mouse lung cells were suspended in FACS buffer. After 5-min incubation with Purified Rat Anti-Mouse CD16/CD32, cells were incubated with a cocktail of monoclonal antibodies, which included anti-mouse CD45 FITC (Thermo Fisher Scientific, Waltham, MA), BV421 anti-mouse F4/80, and PE/Cy7 anti-mouse CD11c antibody (BioLegend, San Diego, CA). As isotype controls of those antibodies, rat IgG2b κ isotype control FITC (Thermo Fisher Scientific, Waltham, MA), PE/Cy7 Armenian hamster IgG isotype control and BV421 rat IgG2a, κ isotype control (BioLegend, San Diego, CA) were used.

Flow cytometry was performed using a FACS Verse (BD Biosciences, San Jose, CA) and the FlowJo software program (Treestar).

### 2.7. Harvesting bronchoalveolar cells

Bronchoalveolar cells were harvested as described below (Uemura et al., 2017). One day after the administration of LNA-ASOs, mice were sacrificed by exsanguination under anesthesia with 2.5% isoflurane, and bronchoalveolar lavage fluid (BALF) samples were collected by making an incision in the trachea and washing the lungs twice with 0.75 mL PBS. BALF samples from each mouse were centrifuged at 2000 rpm for 2 min at 4 °C. Cell pellets were collected as bronchoalveolar cells.

### 2.8. Quantitative reverse transcription polymerase chain reaction (RT-PCR)

After culturing the thioglycollate-elicited murine peritoneal macrophages with LNA-ASOs, total RNA was extracted using a Maxwell RSC simplyRNA Cells Kit (Promega, Madison, WI) and MaxWell RSC (Promega, Madison, WI). Bronchoalveolar cells, lung, liver, and kidney from PBS or LNA-ASOs-treated mice were homogenized using a homogenization buffer, Buffer RLT (QIAGEN, Hilden, Germany) with dithiothreitol solution (Nakalai Tesque, Kyoto, Japan), and a tissue lyser II (QIAGEN, Hilden, Germany). Total RNA was extracted from the lysate using a Maxwell RSC simplyRNA Cells Kit or Maxwell RSC simplyRNA Tissue Kit and MaxWell RSC (Promega, Madison, WI). cDNA was synthesized using SuperScript VILO, according to the manufacturer’s protocol. mRNA levels were evaluated by quantitative RT-PCR using TaqMan Fast Universal PCR Master Mix (Thermo Fisher Scientific, Waltham, MA) with QuantStudio 12K flex and the following TaqMan Probes (Thermo Fisher Scientific, Waltham, MA): mouse Scarb1, Mm00450234_m1; mouse Hprt1, Mm03024075_m1; mouse Actb, Mm00607939_s1; mouse G-CSF, Mm00438334_m1; mouse Cxcl1, Mm04207460_m1; mouse IL-6, Mm00446190_m1; and mouse TNF-α, Mm00443258_m1. The relative mRNA expression was quantified using the comparative CT method.

### 2.9. Statistical analyses

The statistical significance of a KD effect *in vitro* was calculated by the Williams test. The statistical significance of the KD or immune response was calculated by the Wilcoxon rank sum test using the SAS software (SAS Institute, Cary, NC) program. *p* Values of <.05 were considered to indicate statistical significance.

## 3. Results

### 3.1. The uptake and KD effect of LNA-ASOs in thioglycollate-elicited murine peritoneal macrophages

**Figure 1.**
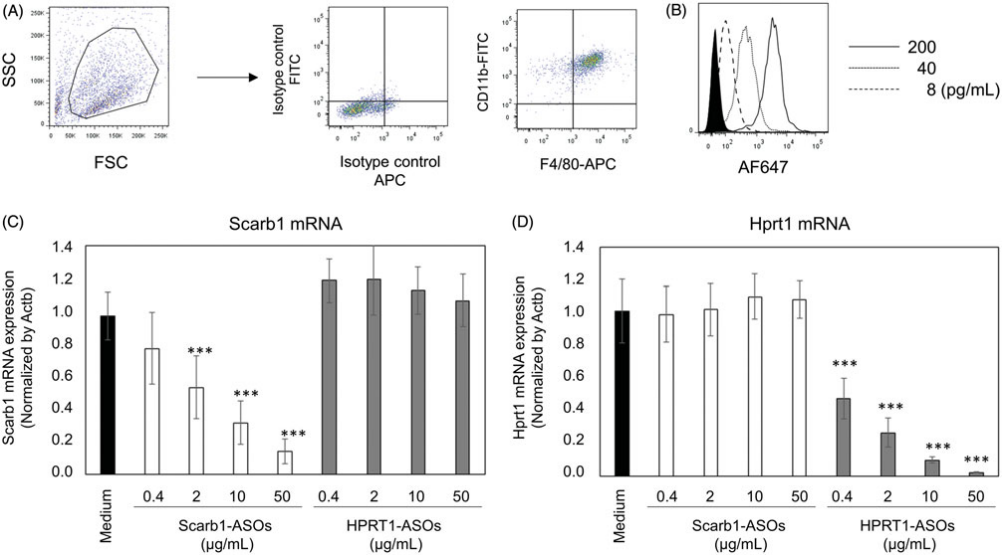
The uptake and KD effect of LNA-ASOs in murine peritoneal macrophages *in vitro*. Thioglycollate-elicited murine peritoneal macrophages were harvested and the expression of CD11b and F4/80 was analyzed using flow cytometry (A). Macrophages were co-cultured with 8, 40, or 200 pg/mL of AF647-conjugated Scarb1-ASOs for 2 h. After which, the cells were harvested and analyzed using flow cytometry (B). The filled histogram shows cells cultured with medium. Representative figures are shown from four independent experiments. Thioglycollate-elicited murine peritoneal macrophages were co-cultured with Scarb1-ASOs or Hprt1-ASOs. After one-day culture, the expression of Scarb1 mRNA (C) or Hprt1 mRNA (D) was measured. The values represent the mean ± SD of four independent experiments. ***p<.001 versus the Medium group (Williams’ test).

To investigate the uptake and KD effect of LNA-ASOs in murine macrophages, murine peritoneal cells were prepared from thioglycollate-treated mice. Adhered thioglycollate-elicited peritoneal cells were CD11b + F4/80+ macrophages (Figure 1(A)). AF647-conjugated Scarb1-ASOs were cultured with those macrophages for 2 h. After this culture, macrophages were harvested and analyzed by flow cytometry. The fluorescence intensity of AF647 was concentration dependently increased by incubation of AF647-conjugated Scarb1-ASOs (Figure 1(B)).

Subsequently, to determine a KD effect of LNA-ASOs in murine macrophages *in vitro*, thioglycollate-elicited murine peritoneal macrophages were cultured with 0.4, 2, 10, or 50 µg/mL of Scarb1-ASOs or Hprt1-ASOs. In comparison to the medium-treated cells, Scarb1-ASOs significantly and concentration dependently suppressed Scarb1 mRNA without suppressing the Hprt1 mRNA expression (Figure 1(C)). In addition, Hprt1-ASOs also induced a significant and concentration-dependent KD, without suppressing the Scarb1 mRNA expression (Figure 1(D)).

### 3.2. Distribution of intratracheally administered LNA-ASOs in the murine lung

**Figure 2.**
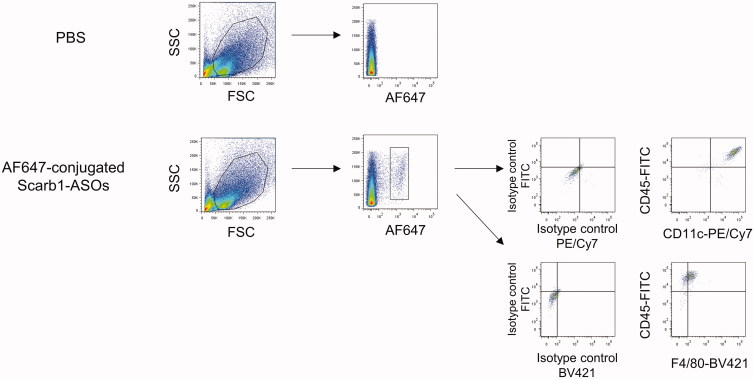
The accumulation of intratracheally administered LNA-ASOs in murine alveolar macrophages. AF647-conjugated Scarb1-ASOs were intratracheally administered to C57BL/6 mice. Two hours later, the lung was collected and AF647-positive cells were detected using flow cytometry. Alveolar macrophages were identified as CD45+, F4/80+, and CD11c+.

To analyze the distribution of intratracheally administered LNA-ASOs in the murine lung, AF647-conjugated Scarb1-ASOs were intratracheally administered. Two hours after administration, primary lung cells were harvested and analyzed by flow cytometry. AF647-positive cells were observed in mice treated with AF647-conjugated Scarb1-ASOs (Figure 2). These cells were CD45+, F4/80+, and CD11c + alveolar macrophages.

### 3.3. The KD effect of the intratracheal administration of LNA-ASOs in alveolar macrophages

**Figure 3.**
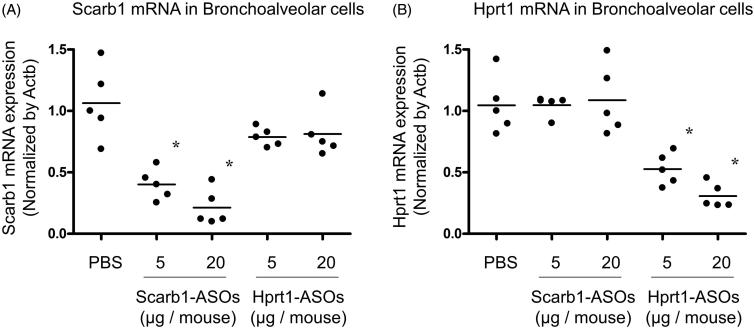
The KD effect of Scarb1-ASOs and Hprt1-ASOs in murine alveolar macrophages *in vivo*. Scarb1-ASOs or Hprt1-ASOs were intratracheally administered to C57BL/6 mice. One day after the administration, bronchoalveolar cells were collected, and the expression of Scarb1 mRNA (A) and Hprt1 mRNA (B) was measured. The dots indicate each measurement in mice (*n* = 5). Horizontal bars indicate the mean values. *p<.05 vs. the PBS group (Wilcoxon’s rank sum test).

Based on the observation that intratracheally administered AF647-conjugated LNA-ASOs were most abundant in alveolar macrophages, it was hypothesized that the intratracheal administration of LNA-ASOs effectively exerted a KD effect in alveolar macrophages. Scarb1-ASOs or Hprt1-ASOs (5 or 20 µg per animal) were intratracheally administered to mice using a MicroSprayer. One day after administration, bronchoalveolar cells, which were mainly composed of alveolar macrophages (Kurotaki et al., 2013; Park et al., 2013), were harvested and total mRNA was extracted. Compared to PBS-treated mice, the expression of scarb1 mRNA in mice that received 5 µg of Scarb1-ASOs was reduced by 62%, while that of mice that received 20 µg of Scarb1-ASOs was reduced by 80% (Figure 3(A)). This suppression was significant and dose-dependent. Hprt1-ASOs treatment did not suppress the scarb1 mRNA expression. Compared to the PBS-treated group, the hprt1 mRNA expression of mice that received 5 µg of Hprt1-ASOs was reduced by 47%, while that of mice that received 20 µg of Hprt1-ASOs was reduced by 70% (Figure 3(B)). Scarb1-ASOs did not suppress hprt1 mRNA. These results indicated that the intratracheal administration of each LNA-ASO exerted sequence-dependent KD in murine alveolar macrophages.

### 3.4. The KD effect of the intratracheal administration of LNA-ASOs in the whole lung

**Figure 4.**
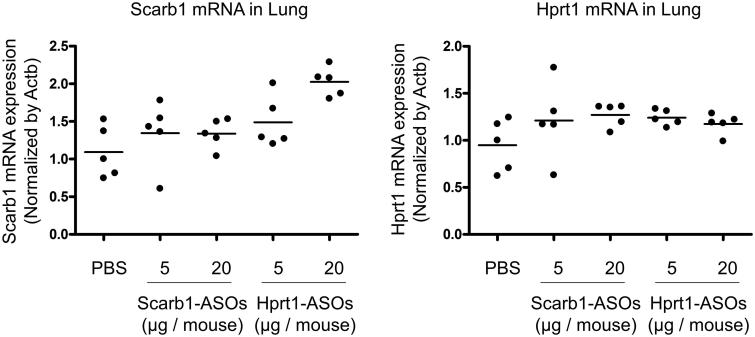
The KD effect of Scarb1-ASOs and Hprt1-ASOs in the murine whole lung *in vivo*. Scarb1-ASOs or Hprt1-ASOs were intratracheally administered to C57BL/6 mice. One day after the administration, the lung was collected, and the expression of Scarb1 mRNA (A) and Hprt1 mRNA (B) was measured. The dots indicate each measurement in mice (*n* = 5). Horizontal bars indicate the mean values.

In our previous report, we confirmed a KD effect of the intratracheal administration of Scarb1-ASOs in the whole lung. The dose required for KD (50–64%) was 40 or 100 µg/mouse, once daily for two days (Uemura et al., 2017). These doses were much higher than the dose required for alveolar macrophages. We therefore considered that the dose required for KD in alveolar macrophages might not be sufficient for KD in the whole lung. To confirm this, whole lungs were collected one day after the intratracheal administration of LNA-ASOs, and a KD effect was evaluated. The target mRNA expression was not suppressed by Scarb1-ASOs or Hprt1-ASOs (Figure 4(A,B)).

### 3.5. The effect of LNA-ASOs on the immune response in the whole lung

In our previous report, when more than 40 μg of Scarb1-ASOs was administered once daily for two days, a KD effect was observed in the lung, while the expression of Cxcl1 and G-CSF mRNA was markedly increased (approximately 10- to 50-fold) in comparison to the solvent-treated group. As the dose required for a KD effect in alveolar macrophages was lower than that for the whole lung, it was hypothesized that the risk of an immuno-stimulatory effect would be consequently reduced. To test this hypothesis, whole lungs were harvested one day after the intratracheal administration of LNA-ASOs, and the expression of inflammatory cytokine mRNA was analyzed. As macrophages are known to be the source of TNF-α and IL-6 (Atri et al., 2018), in addition to the Cxcl1 and G-CSF, these cytokines were also evaluated.

**Figure 5.**
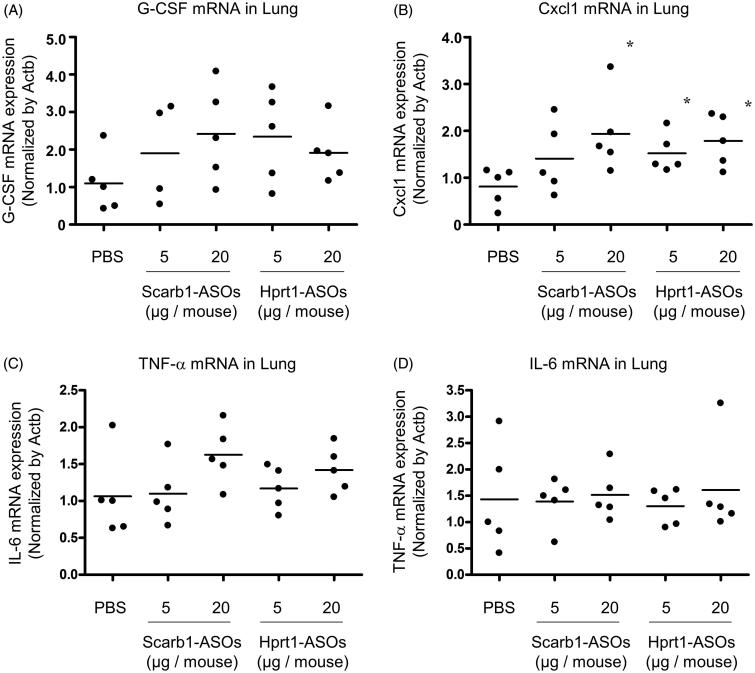
The effect of Scarb1-ASOs and Hprt1-ASOs on the inflammatory cytokine mRNA expression in the lungs. Scarb1-ASOs or Hprt1-ASOs were intratracheally administered to C57BL/6 mice. One day after the administration, the lung was collected, and the expression of G-CSF (A), Cxcl1 (B), TNF-α (C), and IL-6 (D) mRNAs was measured. The dots indicate each measurement in mice (*n* = 5). Horizontal bars indicate the mean values. *p<.05 by Wilcoxon’s rank sum test.

In the whole lung, an approximately twofold increase in the expression of G-CSF and Cxcl1 mRNA was observed in each of the LNA-ASOs treated groups in comparison to the PBS-treated group. The Cxcl1 mRNA expression was significantly increased in mice that received 20 μg of Scarb1-ASOs and 5 or 20 μg of Hprt1-ASOs (Figure 5(A,B)). TNF-α mRNA expression tended to increase in the mice that received 20 μg of Scarb1-ASOs (an approximately 1.5-fold increase) and those that received 20 μg of Hprt1-ASOs (an approximately 1.3-fold increase) in comparison to the PBS-treated group (Figure 5(C)). Regarding the IL6 mRNA expression, no increase was observed with any of the LNA-ASOs (Figure 5(D)).

### 3.6. The KD effect of the intratracheal administration of LNA-ASOs in the liver and kidney

**Figure 6.**
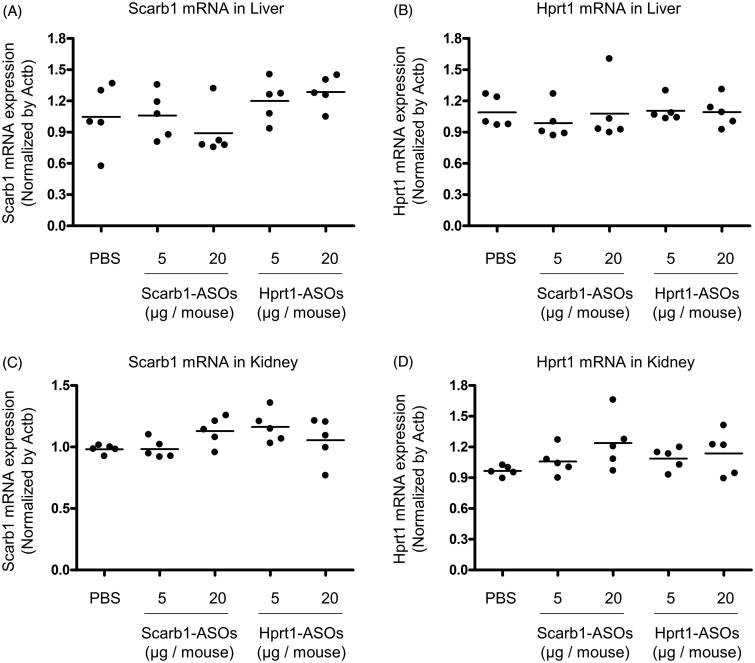
The KD effect of Scarb1-ASOs and Hprt1-ASOs in murine liver and kidney *in vivo*. Scarb1-ASOs or Hprt1-ASOs were intratracheally administered to C57BL/6 mice. One day after the administration, the liver (A and B) and kidney (C and D) were collected and the expression of Scarb1 mRNA and Hprt1 mRNA was measured. The dots indicate each measurement in mice (*n* = 5). Horizontal bars indicate the mean values.

Since the lung is well known to be permeable to small molecules and the systemic administration of ASOs exerts a KD effect on the liver and kidney (Swayze et al., 2007; Donner et al., 2015; Shen & Corey, 2018), intratracheally administered LNA-ASOs may leak out of the lungs and exert their KD effect on those organs. To test this possibility, Scab-1-ASOs or Hprt1-ASOs were intratracheally administered to mice at the dose that induced KD in alveolar macrophages. One day after the intratracheal administration, the liver and kidney were collected and a KD effect was evaluated. No KD effect was observed in any LNA-ASO-treated mice (Figure 6(A–D)). The mRNA expression of scarb1 and hprt1 was similar among the lung, liver, and kidney. The quantitative RT-PCR cycle threshold values of scarb1 and hprt1 were approximately 22–24 in these organs.

## 4. Discussion

The present study confirmed the remarkable accumulation of intratracheally administered LNA-ASOs in murine alveolar macrophages (Figure 2), which showed a significant KD effect that was not observed in the whole lung (Figures 3 and 4). However, several inflammatory cytokines were increased in the whole lung (Figure 5). In addition, when LNA-ASOs were intratracheally administered at a dose that induced KD in alveolar macrophages, no KD was observed in the liver or kidney (Figure 6).

In the present study, as experimental tools, two types of LNA-ASOs targeting Scarb1 or Hprt1 mRNA were prepared. *In vitro*, these LNA-ASOs were accumulated in primary thioglycollate-elicited murine peritoneal macrophages and exerted a significant and sequence-dependent KD effect in these cells without the use of a transfection reagent (Figure 1(A,B)). These results suggest that LNA-ASOs have the potential to induce KD in macrophages without any drug delivery system. Both the cellular uptake and endosomal escape are recognized as important factors for inducing a KD effect of ASOs (Crooke et al., 2017). Macrophages are expected to take up ASOs effectively because they have a high phagocytic ability. However, it is well known that substances taken up by macrophages are rapidly degraded in lysosomes (Weiss & Schaible, 2015), so such a mechanism may reduce LNA-ASOs’ endosomal escape efficiency. Unfortunately, no information is available concerning the endosomal escape of LNA-ASOs using phagocytes. To improve the potency of LNA-ASOs in macrophages, further investigation of this issue is required.

As alveolar macrophages have been reported to be involved in various pulmonary diseases (Opitz et al., 2010), the regulation of such cells by LNA-ASOs would be helpful for the development of novel therapies. Alveolar macrophages are present in the alveolar space and have the property of taking up foreign antigens (Hussell & Bell, 2014; Kopf et al., 2015; Morales-Nebreda et al., 2015), thus, they are presumed to be the most suitable target for inhaled LNA-ASOs. In the present study, we investigated the distribution of intratracheally administered fluorescence-labeled LNA-ASOs in murine lung and found that the accumulation was the most conspicuous in alveolar macrophages (Figure 2). Therefore, targeting alveolar macrophage is presumed to allow for KD at low doses. Indeed, it was confirmed that the intratracheal administration of LNA-ASOs exerted a significant and sequence-dependent KD effect in bronchoalveolar cells (Figure 3(A,B)), which were mainly composed of alveolar macrophages (Kurotaki et al., 2013; Park et al., 2013). The dose required for KD (approximately 50–80%) was a single injection of 5 or 20 µg per mouse, which was too low to induce KD in the whole lung (Figure 4(A,B)). On the other hand, in our previous study, the dose required for KD (50–65%) in the whole lung was 40 or 100 µg per mouse, once daily for two days (Uemura et al., 2017). These results indicated that the dose required for KD in alveolar macrophages was much lower than that required for KD in the whole lung.

As described above, the dose required for KD in alveolar macrophages was lower than that required for KD in the whole lung. We therefore expect the risk of immuno-stimulation to be lower than if the whole lung had been targeted. In our previous report, the intratracheal administration of Scarb1-ASOs at a dose sufficient to exert KD in approximately 50–65% of the whole lung resulted in an approximately 10- to 50-fold increase in the expression of G-CSF and Cxcl1 mRNA compared to the expression after solvent treatment (Uemura et al., 2017). In contrast, in the present study, when 50–80% KD was observed in alveolar macrophages, the expression of G-CSF and Cxcl1 mRNA in the lung was increased approximately twofold compared to the expression after solvent treatment (Figure 5(A,B)). The present results suggest that targeting alveolar macrophages by inhaled LNA-ASOs carries a lower risk of immuno-stimulation than targeting the whole lung.

However, the expression of Cxcl1 mRNA was significantly increased, and the expression of G-CSF and TNF-α mRNA in the lung tended to increase after the intratracheal administration of Scarb1-ASOs or Hprt1-ASOs at doses of 5 or 20 µg (Figure 5(A–C)). Therefore, the risk of an immuno-stimulatory effect was not completely avoided. There are various cells in the lung that can trigger an inflammatory response (e.g. alveolar macrophages, dendritic cells, lung epithelial cells). However, which cells are the cause of the inflammation induced by the intratracheal administration of LNA-ASOs is unclear (Uemura et al., 2017). The further investigation of this issue will be required to eliminate the immuno-stimulatory risk associated with LNA-ASOs. Regarding active targeting, it was reported that N-acetylgalactosamine-conjugated ASOs have a significantly improved KD effect in the liver (Prakash et al., 2014). Therefore, conjugating a DDS element targeting the endogenous receptor of macrophages may improve the KD potency and help reducing the risk of immuno-stimulation.

In case of lung-specific treatment, the leakage of LNA-ASOs from the lung is an issue because the lung is permeable to small molecules. Indeed, pulmonary administration has even been attempted in an effort to achieve the whole-body exposure of an agent (insulin) (Kim & Plosker, 2015; Chan & Cheng-Lai, 2017). In addition, it is also known that ASOs exert KD in the liver and kidney when administered systemically (Swayze et al., 2007; Donner et al., 2015; Shen & Corey, 2018). In our experiment, a KD effect was not observed in those organs under doses that induced 50–80% KD in alveolar macrophages. These results suggest that the inhalation of LNA-ASOs at low doses has the potential to exert a KD effect that is specific to alveolar macrophages.

In conclusion, we confirmed that intratracheally administered LNA-ASOs remarkably accumulated in alveolar macrophages and exerted a significant and sequence-dependent KD effect. The dose that was sufficient to induce a KD effect in alveolar macrophages was lower than that required to induce KD in the whole lung. However, an inflammatory response was still observed in the lung. The dosage that induced a KD effect in alveolar macrophages did not induce a KD effect in the liver or kidney. In the present study, LNA-ASOs showed the potential utility as an inhaled drug specific to alveolar macrophages. However, the further study of the immuno-stimulatory effects of LNA-ASOs will be necessary in order to develop safe and effective inhaled LNA-ASOs therapies.
